# Does access to a demand-led evidence briefing service improve uptake and use of research evidence by health service commissioners? A controlled before and after study

**DOI:** 10.1186/s13012-017-0545-4

**Published:** 2017-02-14

**Authors:** Paul M Wilson, Kate Farley, Liz Bickerdike, Alison Booth, Duncan Chambers, Mark Lambert, Carl Thompson, Rhiannon Turner, Ian S Watt

**Affiliations:** 1 Alliance Manchester Business School, University of Manchester, Booth Street East, Manchester, M15 6PB UK; 2 School of Healthcare, University of Leeds, Leeds, UK; 3 Centre for Reviews and Dissemination, University of York, York, UK; 4 York Trials Unit, University of York, York, UK; 5 School of Health and Related Research, University of Sheffield, Sheffield, UK; 6 Public Health England North East Centre, Newcastle upon Tyne, UK; 7 School of Psychology, Queen’s University Belfast, Belfast, UK; 8 Department of Health Sciences, University of York, York, UK

## Abstract

**Background:**

The Health and Social Care Act mandated research use as a core consideration of health service commissioning arrangements in England. We undertook a controlled before and after study to evaluate whether access to a demand-led evidence briefing service improved the use of research evidence by commissioners compared with less intensive and less targeted alternatives.

**Methods:**

Nine Clinical Commissioning Groups (CCGs) in the North of England received one of three interventions: (A) access to an evidence briefing service; (B) contact plus an unsolicited push of non-tailored evidence; or (C) unsolicited push of non-tailored evidence. Data for the primary outcome measure were collected at baseline and 12 months using a survey instrument devised to assess an organisations’ ability to acquire, assess, adapt and apply research evidence to support decision-making. Documentary and observational evidence of the use of the outputs of the service were sought.

**Results:**

Over the course of the study, the service addressed 24 topics raised by participating CCGs. At 12 months, the evidence briefing service was not associated with increases in CCG capacity to acquire, assess, adapt and apply research evidence to support decision-making, individual intentions to use research findings or perceptions of CCG relationships with researchers. Regardless of intervention received, participating CCGs indicated that they remained inconsistent in their research-seeking behaviours and in their capacity to acquire research. The informal nature of decision-making processes meant that there was little traceability of the use of evidence. Low baseline and follow-up response rates and missing data limit the reliability of the findings.

**Conclusions:**

Access to a demand-led evidence briefing service did not improve the uptake and use of research evidence by NHS commissioners compared with less intensive and less targeted alternatives. Commissioners appear well intentioned but ad hoc users of research. Further research is required on the effects of interventions and strategies to build individual and organisational capacity to use research.

## Background

In the National Health Service (NHS) in England, Clinical Commissioning Groups (CCGs) are responsible for the planning and commissioning of health care services in a defined geographical area. In 2012, the Health and Social Care Act mandated research use as a core consideration in health service commissioning arrangements [1]. Each CCG now must, in the exercise of their functions, promote the use in the health service evidence obtained from research [1].

NHS commissioners now have a key role in improving the uptake and use of knowledge to inform commissioning and decommissioning of services, and there is a substantive evidence base upon which they can draw. However, uptake of this knowledge to increase efficiency, reduce practice variations and ensure best use of finite resources within the NHS is not always realised. This is in part through system failings to fully implement interventions and procedures of known effectiveness [2, 3]. There has also been rapid, sometimes policy-driven deployment of unproven interventions despite known uncertainties relating to costs, impacts on service utilisation and clinical outcomes, patient experience and sustainability [4]. And the NHS has been slow to identify and disinvest in those interventions known to be of low or no clinical value [5].

Whilst it is widely acknowledged that different sources of knowledge combine in evidence-informed decision-making [6] and that the process itself is highly contingent and context dependent [7], the value of systematic reviews to health care decision-making is well recognised [8, 9]. However, a number of challenges have undermined the usefulness of systematic reviews in decision-making contexts [8, 10–15]. These include difficulties in locating and appraising relevant reviews, a lack of timeliness or user friendliness and a perceived failure of reviews to address relevant questions, contextualize the findings or make actionable policy recommendations. An initiative aiming to enhance uptake of systematic review evidence by NHS commissioners and senior managers was developed as an adjunct to the implementation theme of the NIHR CLAHRC for Leeds, York and Bradford [16]. Development of the service was informed by a scoping review of existing resources [17] and previous experience in producing and disseminating the renowned Effective Health Care and Effectiveness Matters series of bulletins. The service attempted to inform real decisions by making use of existing sources of synthesised research evidence. The service approach was both consultative and responsive and involved building relations and having regular contact (face to face and email) with a range of NHS commissioners and managers. This enabled the team to discuss issues and for those that required a more considered response, formulate questions from which contextualised briefings could be produced and their implications discussed. In doing so, we utilised a framework designed to clarify the problem and frame the question to be addressed [18]. The service had some early impacts notably including work to inform service reconfiguration for adolescent eating disorders; enabling commissioners to invest in more services on a more cost-effective outpatient basis [19].

Although feedback from users was consistently positive, the evidence briefing service had been developmental and no formal evaluation had been conducted. The service as constituted was a resource-intensive endeavour and made use of the considerable review capacity and infrastructure available at the Centre for Reviews and Dissemination (CRD). As such, this study aimed to assess whether access to a demand-led evidence briefing service would improve the uptake and use of research evidence by NHS commissioners compared with less intensive and less targeted alternatives.

## Methods

This was a controlled before and after study involving CCGs in the North of England [20]. The study protocol has been published previously [21].

### Setting, participants and recruitment

**Fig. 1:**
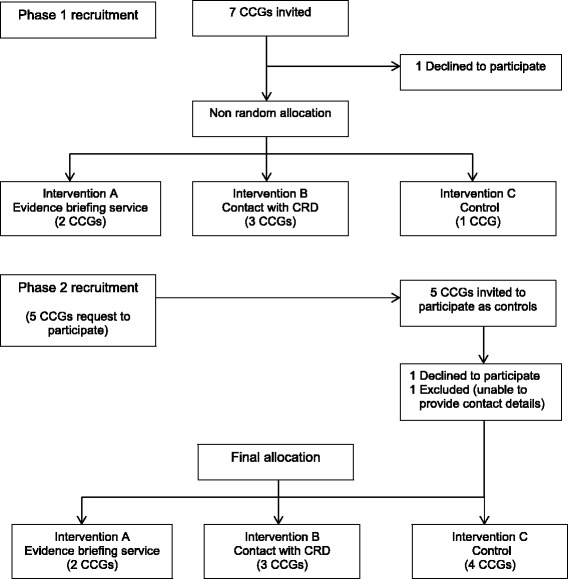
Flow diagram of CCG recruitment

Nine CCGs from one geographical area in England agreed to participate, and the recruitment process is presented in Fig. 1. We had originally anticipated that we would invite 9–10 CCGs from one geographical area based on the 2012/13 Primary Care Trust (PCT) cluster arrangements. By the start of the study, some consolidation in the proposed commissioning arrangements had occurred in the transition from PCTs to CCGs and so seven CCGs were invited to participate. Of these, six agreed to participate. One CCG declined, intimating that they could not participate in any intervention. No CCG asked for financial reimbursement for taking part in the study.

We had originally intended to randomly allocate CCGs to interventions. However, a combination of expressed preferences (one CCG indicated that they would like to be a ‘control’) and the prospect of further consolidation in commissioning arrangements meant that this was not feasible. Taking these factors into account, two CCGs were allocated to receive on-demand access to the evidence briefing service, three coterminous CCGs (who were likely to merge) received on-demand access to advice and support from the CRD team and one to a ‘standard service’ control arm. After this initial allocation, research leads from CCGs in a neighbouring geographical area approached the team and asked to participate. After discussions with representatives of the five CCGs, a further three CCGs were recruited as ‘standard service’ controls.

### Interventions

Participating CCGs received one of three interventions aimed at supporting the use of research evidence in their decision-making:

A. Contact plus responsive push of tailored evidence
  CCGs in this arm received on-demand access to an evidence briefing service provided by research team members at CRD. In response to questions and issues raised by a CCG, the CRD team would synthesise existing evidence together with relevant contextual data to produce tailored evidence briefings to a specified timescale agreed with the CCG. We anticipated responding to six to eight substantive issues per CCG during the intervention phase. This was a responsive service, and CCGs could contact the intervention team at any time to request their services. Contact initiated by the CRD intervention team was made on a monthly basis and was expected to include discussion of questions and priority topics and offers of advice and support around identifying, appraising and interpreting evidence. A full account of the service offered is available elsewhere [20].

B. Contact plus an unsolicited push of non-tailored evidence
  CCGs allocated to this arm received on demand access to advice and support from CRD as those allocated to receive on demand access to the evidence briefing service. However, the CRD intervention team did not produce evidence briefings in response to questions and issues raised but instead disseminated the evidence briefings generated in the responsive push intervention.

C. ‘Standard service’ unsolicited push of non-tailored evidence
  The third intervention constituted a ‘standard service’ control arm; thus, an unsolicited push of non-tailored evidence. In this, CRD used email- and web-based communication processes to disseminate evidence briefings generated in intervention A and any other non-tailored briefings produced by CRD over the intervention period.
  The intervention phase ran from the end of April 2014 to the beginning May 2015. As this study was evaluating uptake of a demand-led service, the extent to which the CCGs engaged with the interventions was determined by the CCGs themselves.

### Baseline and follow-up assessment

We collected data for our two primary outcome measures (perceived organisational capacity to use research evidence and reported research use) at baseline (phase 1) and again 12 months after the intervention period was completed (phase 3).

The survey instrument has been published and described previously [21]. The instrument was designed to collect four sets of information that assessed: the organisations’ ability to acquire, assess, adapt and apply research evidence to support decision-making; the intentions of individual CCG staff to use research evidence in their decision-making; perceptions of the quality and quantity of interactions with researchers; and captured information on individual respondent characteristics.

### Survey administration

Each participating CCG supplied a list of names and email addresses for potential respondents. These were checked by a member of the evaluation team, and where inaccurate or missing details were identified, these were sourced and corrected. Survey instruments were sent by personalised email to identified participants via an embedded URL. The questionnaire was hosted by SurveyMonkey website (http://www.surveymonkey.com). Reminder emails were sent out to non-respondents at 2, 3 and 4 weeks. A paper version was also posted out, and phone call reminders were made by the research team. In addition, the named contact in each CCG sent an email to all their colleagues encouraging completion.

As CCGs were new and evolving entities at the time of the study, we needed to be able to determine if any changes viewed from baseline were linked to the intervention(s) and were not just a consequence of the development of the CCG(s) over the course of the study. To guard against this maturation effect/bias and to test the generalisability of findings, we administered Section A of the instrument to all English CCGs to assess their organisational ability to acquire, assess, adapt and apply research evidence to support decision-making. The most senior manager (chief operating officer or chief clinical officer) of each CCG was contacted and asked to complete the instrument on behalf of their organisation. For the national survey, we used publically available information (NHS England and CCG websites) supplemented by phone calls to CCG headquarters to construct our sampling frame consisting of every CCG in England.

### Analysis

The primary analysis measured the effect of study interventions on two main outcomes at two time points: baseline and 12 months. The key dependent variable was CCG perceived organisational capacity to use research evidence in their decision-making as measured by Section A of the survey instrument. Following Norman [22], we calculated the means, standard deviations and confidence intervals for each of the seven subscales in the instrument before and after the intervention period; CCG capacity to acquire research, to look for research in the right places, to tell if research is valid and of high quality, to tell if research is relevant and applicable, to summarise results in a user-friendly way, to lead by example and value research use to support decision-making processes for research use. We also calculated an overall mean—of CCG capacity—and standard deviation (mean of the sub scale means). Interpreting the means is similar to the original scale: a mean score nearer to 1 equates to very little capacity and a mean score nearer to 5 indicates good capacity in the respective sub domain or for research evidence use generally.

We also measured the effect of interventions upon our second main outcome of perceived research use and CCG member’s intentions to use research. Based on the theory of planned behaviour [23], we calculated means for the four subscales in our survey instrument: intention to use research, attitudes towards research, group norms and perceived behavioural control. The Likert scale used ranged from a score of 1, indicating the lowest amount of the concept being measured (e.g. no intention to use research) through to a score of 7 indicating the highest amount of the concept being measured (e.g. the most positive attitudes towards research use in a CCG).

We undertook a factorial ANOVA (SPSS version 22.0 general linear model procedure), comparing the main effect of a single independent variable (CCG status) on a dependent variable (capacity to acquire, assess, adapt and apply research evidence to support decision-making) ignoring all other independent variables (i.e. the effect ignoring the potential for confounding from other independent factors). A factorial ANOVA was also conducted to compare the main effects of time and evidence briefing service received and the interaction effect of time and evidence briefing on intention to use research evidence (the ‘intention’ component of the survey instrument).

To examine the effects of (i) perceived contact and (ii) the amount of perceived contact with the evidence briefing service, (iii) institutional support for research, (iv) a sense of being equal partners during contact, (v) common in-group identity, (vi) achievement of goals, and (vii) perceptions of researchers generally, we undertook a mixed 3 (intervention, A vs. B vs. C) × 2 (time, baseline vs. outcome) ANOVA using SPSS version 22.0, with the intervention as a between-subject independent variable, and repeated measures on the second factor, time.

### Missing data

Only analysing the data for which we had complete responses could lead to potentially biased results [24], and as anticipated at the protocol stage, the use of multiple imputation techniques were required [25]. We assumed that data were missing at random (visual comparison of original vs. imputed data and significance testing of response and nonresponse data impact on outcome variables). We used guidance on interpreting effect sizes in before and after studies to examine the clinical/policy significance of any changes [26].

### Blinding

The CRD evidence briefing team were blinded from both baseline and follow-up assessments until after all the data collection was complete. The CRD team were made aware of baseline and follow-up response rates. Participating CCGs were also blinded from baseline and follow-up assessments and analysis.

### Qualitative evaluation

Part of our original plan was to collect and analyse documentary evidence of the actual use of evidence in decision-making using executive and governing body meeting agendas, minutes and associated documents. This was to be supplemented with interviews to explore perceived use of evidence and any unanticipated consequences. Early in the intervention phase, it became apparent that with a few exceptions, there was a lack of recorded evidence of research use (a finding in itself). Executive and governing body meetings were mainly used to ratify recommendations and so would not tell us anything about sources or processes. With research use and decisions occurring elsewhere and often involving informal processes, we undertook four case studies to explore the use of research evidence in decision-making in the intervention sites. A full account of case study methods and analysis are available elsewhere [20].

## Results

Over the course of the study, we addressed 24 questions raised by the participating CCGs, 17 of which were addressed during the intervention phase (see Table 1). The majority of requests were focussed on options for the delivery and organisation of a range of services and way of working rather than on the effects of individual interventions.

**Table 1:** Questions addressed by the evidence briefing service

Source	Topic	Question	Date asked	Output produced	Way research used
A1	Urgent care services	Evidence for implementing an ‘urgent care hub’, consolidating out-of-hours provision on a single site adjacent to an accident and emergency department, with front door triage assessing patients for both facilities	Nov 2013	Evidence briefing	Symbolic
A1	Supporting self-management: helping people manage long-term conditions	Rapid summary of the evidence relating to self-care	Jan 2014	Evidence note	Symbolic
All	Urgent care services	Evidence to inform urgent and emergency care systems	March 2014	Evidence briefing	Conceptual
A1	Loneliness and social isolation	Interventions to reduce loneliness and social isolation, particularly in elderly people.	Apr 2014	Evidence briefing	Conceptual
A1	Supporting self-management: helping people manage long-term conditions	Self-care support for people with COPD	Apr 2014	Evidence briefing	Conceptual
All	Low value interventions	Identify relevant recommendations from the NICE Do Not Do database	May 2014	Evidence note	Conceptual
A2, all	Low value interventions	Independent appraisal of evidence underpinning 14 proposed value based commissioning policies for MSK procedures	July 2014	Evidence briefing	Instrumental
A1	Community pharmacy minor ailments service	Identify evidence to inform a review of the community pharmacy minor ailments service	July 2014	Evidence note	Conceptual
A1	Integrated community teams	Evidence for effects of integrated community teams including any examples of best practice	Aug 2014	Evidence note	Conceptual
A2	Psychiatric Liaison	Models of psychiatric liaison implemented in general hospital settings	July 2014	Evidence note	Instrumental
A1	‘One stop shop’ screening model for diabetes	Does implementing a comprehensive one stop shop annual review and screening model for diabetes have an adverse impact on either the quality or uptake	Sept 2014	Evidence note	Symbolic
A2	Frailty	What evidence/ validated tools are there for frailty risk profiling in an A&E context	Oct 2014	Short email Note sufficient to address question. Later followed up with related *Effectiveness Matters* on recognising and managing frailty in primary care	Conceptual
A2	Unplanned admissions from care homes	What is the evidence for effects of interventions to reduce inappropriate admissions and deaths in hospital of patients from care homes	Oct 2014	Evidence briefing	Conceptual
A2	Social prescribing	What is the effectiveness and cost effectiveness evidence of social prescribing programmes in primary care	Oct 2015	Evidence note and then later updated into evidence briefing	Conceptual
A1	Supporting self-management: helping people manage long-term conditions	What is the evidence for the effects of phone apps to help people to manage their own care	Nov 2015	Evidence note	Instrumental
A1	Supporting self-management: helping people manage long-term conditions	What is the evidence for the effects of interventions to promote shared decision-making	Nov 2015	Evidence note	Conceptual
A1	Supporting self-management: helping people manage long-term conditions	What is the evidence for interventions to support promoting patient-centred care planning consultations	Nov 2015	Evidence briefing	Conceptual
A1	Supporting self-management: helping people manage long-term conditions	Evidence for lay-led self-care education programmes generally as part of creating an environment and culture that supports self-care	Nov 2015	Evidence briefing	Conceptual
A1	Supporting self-management: helping people manage long-term conditions	An evidence based steer in how to give patients the confidence and skills to effectively self-manage their long-term conditions.	Nov 2015	Evidence briefing	Conceptual
A2	Accountable care organisations	What is the evidence for accountable care organisations	Apr 2015	Evidence note	Conceptual
A2	Enhancing access in primary care	What is the evidence for extended hours, telephone consultation/triage, and role substitution in enhancing access in primary care	June 2015	Evidence briefing	Conceptual
A2	Telehealth for COPD	What lessons can be learned from previous evaluations of the implementation of telehealth for COPD	July 2015	Evidence note	Instrumental
A1	Participatory democracy	What is the evidence for different methods of patient/public engagement in decision-making	Aug 2015	Evidence note	Conceptual
All	Low-value interventions: existing hernia and hysterectomy policies	Independent review of evidence for existing hernia and hysterectomy policies	Aug 2015		Instrumental

Requests for evidence briefings from the CCGs served different purposes. Four broad categories of research use have been proposed: conceptual (not directly linked to discrete decisions but to provide knowledge about possible options for future actions); symbolic or tactical (to justify existing decisions and actions); instrumental (where evidence directly informs a discrete decision-making process); and imposed (where there are organisational, legislative or funding requirements that research be used) [27, 28]. Derived through a consensus-based approach, Table 1 shows that most requests received were categorised as conceptual.

### Response rates

Contact details for 181 baseline (A = 45; B = 61; C = 75) and 168 follow-up (A = 43; B = 60; C = 65) participants were supplied by CCGs; none were undeliverable.

In total, 123 questionnaires were returned at baseline (A = 37/45; B = 54/61; C = 32/75) giving an overall response rate of 68%. Of these, 101 were completed, 13 were deemed to be incomplete (one section or less completed) and 9 were from individuals declining to participate or indicating they had departed the CCG. At 1 year follow-up, 76 questionnaires were returned (A = 23/43; B = 28/60; C = 25/65) giving an overall response rate of 44%. Of these, 71 were completed, two were deemed to be incomplete (one section or less completed) and three were from individuals declining to participate or indicating they had departed the CCG.

### Characteristics of respondents

Survey respondents reported holding a range of roles within the CCGs. Most respondents were highly qualified but only a minority reported having had prior experience in commissioning or undertaking research (see Table 2). Sites with a lower response rate had a higher proportion of clinically qualified respondents (*X* ^2^ (2, *N* = 53) = 6.15, *p* = .05) but other than this difference, there were no significant differences in the characteristics of the respondents receiving the three interventions.

**Table 2:** Characteristics of survey respondents

		Intervention received		
		A	B	C
		*n*	*n*	*n*
Formal responsibility for doing or managing research in CCG?	Yes, doing and managing	5	2	2
	Yes, managing	3	3	7
	Yes, doing	1	2	0
	Neither	28	35	17
Highest educational achievement?	School level	2	0	0
	Undergraduate	17	27	12
	Master’s degree	14	13	8
	Higher degree	3	2	6
Clinical qualifications?	No	16	8	6
	Yes	21	34	20
Worked as a researcher in an academic context	No	34	42	24
	Yes	5	11	13
Commissioned research	No	29	47	32
	Yes	10	6	5
Been a co-applicant or advisor on a research project	No	30	44	30
	Yes	9	9	7
Been employed as a researcher	No	35	49	32
	Yes	4	4	5

### Overall capacity to acquire, assess, adapt and apply research evidence to support decision-making

The total capacity to acquire, assess, adapt and apply research evidence to support decision-making appeared to improve slightly over time, irrespective of the presence of any intervention (Table 3). The main effect of time in the factorial ANOVA yielded an *F* ratio of *F*(1, 127) = 4.49 *p* < .05 *η*_*p*_^2^ .034, indicating a significant difference over time in all three groups of CCGs total capacity to acquire, assess, adapt and apply research evidence to support decision-making . The main effect of the evidence briefing service received yielded an *F* ratio of *F*(2, 127) = 0.77 *p* = >.5, *η*_*p*_^2^ .012. The interaction of time and the intervention was also not significant yielding an *F* ratio of *F*(2, 127) = 0.213 *p* > .05, *η*_*p*_^2^ .003. Exposure to the intervention had no significant effect on perceived CCG capacity.

**Table 3:** Intervention effects on CCG capacity to acquire, assess, adapt and apply research evidence to support decision-making

Domain	Intervention received											
	A (*n* = 39)				B (*n* = 53)				C (*n* = 38)			
	Baseline		Follow-up		Baseline		Follow-up		Baseline		Follow-up	
	Mean	95% CI	Mean	95% CI	Mean	95% CI	Mean	95% CI	Mean	95% CI	Mean	95% CI
Total	3.24	3.07–3.41	3.32	3.12–3.51	3.14	2.99–3.28	3.31	3.14–3.48	3.26	3.08–3.43	3.42	3.22–3.62
Acquire (staff)	2.95	2.70–3.18	2.91	2.58–3.22	2.84	2.64–3.05	3.02	2.75–3.29	3.29	2.94–3.43	3.03	2.71–3.35
Acquire (sources)	3.21	2.97–3.44	3.36	3.13–3.56	3.13	2.93–3.33	3.35	3.15–3.55	3.15	2.91–3.39	3.34	3.11–3.58
Assess evidence (staff)	3.04	2.8–3.29	3.34	3.09–3.58	3.28	3.07–3.49	3.42	3.22–3.62	3.36	3.12–3.61	3.27	3.03–3.51
Assess evidence (external expertise)	3.41	3.16–3.64	3.57	3.46–3.79	3.28	2.53–2.99	3.41	3.22–3.60	3.15	2.90–3.39	3.51	3.29–3.74
Adapt	3.09	2.82–3.36	3.29	3.04–3.54	2.76	2.53–2.99	3.12	2.91–3.34	3.10	2.83–3.37	3.24	2.98–3.49
Apply (leadership)	3.45	3.25–3.66	3.31	2.93–3.70	3.22	3.05–3.70	3.16	2.83–3.49	3.37	3.16–3.58	3.62	3.23–4.01
Apply (decision-making)	3.53	3.33–3.72	3.46	3.16–3.77	3.44	3.28–3.62	3.43	3.17–3.69	3.43	3.23–3.63	3.72	3.40–4.02

### Did the evidence briefing service improve CCGs’ intentions to use research evidence to support decision-making?

As with the effect of the evidence briefing service on capacity to use research for decision-making, we also wanted to examine the effect on CCG’s collective intention to use research evidence for decision-making. Attitude towards the use of research in decision-making was the strongest of these dimensions, and perceived behavioural control the weakest (Table 4). All intervention groups had apparent small and non-statistically significant declines in almost all of the theory of planned behaviour dimensions from baseline to follow-up. This difference is—in real terms—marginal: the positions were broadly similar before and after encountering the intervention.

**Table 4:** Intervention impact on theory of planned behaviour domains

Theory of planned behaviour domains	Intervention received											
	A (*n* = 39)				B (*n* = 53)				C (*n* = 38)			
	Baseline		Follow-up		Baseline		Follow-up		Baseline		Follow-up	
	Mean	95% CI	Mean	95% CI	Mean	95% CI	Mean	95% CI	Mean	95% CI	Mean	95% CI
Intention	5.61	5.22–6.00	5.41	5.07–5.76	5.31	5.00–5.61	5.42	5.08–5.76	5.72	5.33–6.11	5.59	5.17–6.02
Attitudes	6.23	5.97–6.49	5.85	5.50–6.20	6.23	5.88–6.30	5.91	5.62–6.20	6.28	6.03–6.54	6.22	5.94–6.49
Group norms	5.18	4.85–5.52	4.77	4.24–5.29	5.03	4.77–5.30	5.02	4.60–5.44	5.39	5.02–5.76	5.43	5.08–5.78
Perceived behavioural control	5.01	4.69–5.33	4.85	4.30–5.40	4.95	4.64–5.25	4.36	3.87–4.85	4.85	4.37–5.33	5.07	4.67–5.47

### Did the evidence briefing service improve CCGs’ perceptions of intergroup contact?

Perceptions of contact appeared generally more positive from the start among the respondents receiving the evidence briefing service than in the other intervention groups (Table 5). There were increases in most other dimensions of contact from baseline to follow-up across the groups. None of these reached statistical significance, and the magnitude of these gains appeared a little lower in intervention A than in interventions B and C.

**Table 5:** Intervention impact on perceptions of intergroup contact between CCGs and researchers

Perceived intergroup contact	Intervention received											
	A (*n* = 39)				B (*n* = 53)				C (*n* = 38)			
	Baseline		Follow-up		Baseline		Follow-up		Baseline		Follow-up	
	Mean	95% CI	Mean	95% CI	Mean	95% CI	Mean	95% CI	Mean	95% CI	Mean	95% CI
Amount of contact	1.76	1.2–2.36	2.11	1.82–2.42	1.17	0.65–1.69	1.72	1.45–2.01	1.16	0.70–1.62	1.92	1.67–2.17
Quality of contact	4.60	3.09–6.11	5.66	5.21–6.11	3.19	1.68–4.67	5.96	5.51–6.41	2.89	1.62–4.13	5.61	5.23–5.99
Institutional (CCG) support for contact	4.60	3.45–5.67	5.12	4.48–5.75	2.68	1.63–3.74	4.61	4.01–5.20	2.56	1.63–3.50	4.79	4.26–5.32
Equal status during contact	4.74	3.56–5.96	4.97	4.57–5.30	3.03	1.92–4.13	4.11	3.73–4.48	2.77	1.78–3.75	4.46	4.12–4.79
Common in-group identity	3.88	2.83–4.92	4.44	4.06–4.81	2.68	1.70–3.66	4.34	3.99–4.69	2.60	1.73–3.47	4.54	4.22–4.85

### Did the evidence briefing service improve CCGs’ perceptions of researchers?

Using a ‘feeling thermometer’ measure where participants reported perceptions of researchers on a scale of 0 (very negative) to 100 (very positive). Perceptions of researchers were positive among the respondents receiving intervention A, at baseline, almost at the level of the post-intervention responses across the board.

There was a significant interaction between intervention and time, *F*(2, 57) = 3.29, *p* = .045. Post hoc analyses demonstrate that perceptions of researchers in general were significantly more positive at the follow-up (*M* = 77.20) than at the baseline (*M* = 46.35) in intervention B, (1, 19) = 9.76, *p* = .006. Similarly, perceptions of researchers were also significantly more positive at the outcome (*M* = 78.21) than at the baseline (*M* = 41.25) in ‘control’ intervention C, (1, 23) = 23.72, *p* = .0005. In contrast, there was no change in attitude towards researchers between the baseline (*M* = 67.31) and the outcome (*M* = 72.69) in intervention A, *F*(1, 15) = 0.36*, p* = .56. In sum, the evidence briefing service did not change perceptions of researchers (in general).

## Discussion

In this study, access to an evidence briefing service was not associated with increases in CCG capacity to acquire, assess, adapt and apply research evidence to support decision-making.

Regardless of intervention received, at baseline, participating CCGs indicated that they lacked a consistent approach to their research-seeking behaviours and their capacity to acquire research remained so at follow-up. CCGs were noncommittal (neither agreeing nor disagreeing) on whether they had the capacity to assess the quality, reliability and applicability of research for use in decision-making. This perception remained unchanged at follow-up. There was also no change on perceptions of CCGs capacity to adapt and summarise research results for use in decision-making; neither agreeing nor disagreeing that the CCG had the capacity to do so. Finally, individual’s perceptions that their CCG did not have systems and processes in place to apply research routinely also remained unchanged.

Exposure to the evidence briefing service did not appear to have any effect on individuals’ intentions to use research evidence in decision-making or their perceptions of a shift in collective CCG norms towards the use of research for decision-making. Regardless of intervention received, these measures were positively orientated at baseline and sustained at follow-up.

The respective baseline and follow-up response rates of 68 and 44% are not unreasonable given the number of competing requests for information CCGs routinely are faced with. Our response rates compare favourably with other surveys conducted over the same time period [29, 30] and with a contemporaneous study of the effects of an evidence service on policy makers’ use of research evidence that failed to recruit [31, 32]. However, we acknowledge that we experienced considerable attrition in both baseline and follow-up surveys. In the study case sites, the percentage of individuals completing both surveys ranged from ~60% for those receiving intervention A to ~30% in the CCGs who were allocated to receive intervention C, the non-responsive version of the service. As the turnover of CCG staff was relatively stable over the course of the study, this may represent a degree of selection bias.

We were reliant on the quality of the sampling frames provided by each CCG. We found that contact information provided by CCGs, and especially that sourced for the national benchmarking component of the study, was sometimes inaccurate and or incomplete. As such, each CCG had to be contacted to obtain, check and recheck the contact details of the staff provided.

We utilised an 87-item questionnaire to collect data relevant to the primary outcome, and although all responses were on short scales (no open responses), piloting estimated that it would take participants up to 45 min to complete. We employed a range of factors to increase the odds of response including, prenotification, follow-up contact, online and postal formats, reminder copies, CCG and university sponsorship [33]. We are aware that both shorter questionnaires and financial incentives are also associated with increased response rates [33]. In this instance, it may be that the perceived return for time invested of access to a funded evidence briefing service either immediately or after the intervention phase was complete (the offer made to participants in the ‘control’ intervention C) and was deemed inadequate compensation by some participants. The CCGs allocated to intervention C had expressed initial enthusiasm for participation. However, the lack of any immediate return from or a sufficient relationship with the evidence briefing service over the course of the study may go some way to explaining why those allocated to the ‘control’ had the lowest response rate. Survey length may also have contributed to the lack of completeness in the data collected.

Given these limitations, we have been cautious in our interpretation of any apparent effect of the evidence briefing service on the primary outcome measures. Indeed, we have been careful to avoid the pitfalls of *p* values in assessing whether this study provides evidence ‘for’ or ‘against’ rejection of the null hypothesis [34]. Whilst the statistical tests applied generated some apparent statistical differences beyond that which we would have expected to see by chance, we think appropriate caution is necessary in interpreting the real world significance of what was observed. It would be reasonable to consider a shift of at least one point on any Likert scale as indicative of some effect and anything lower observed (i.e. no shift on the scale) as unlikely to be behaviourally significant.

Analysis undertaken to trace evidence briefings revealed with few exceptions a lack of recorded evidence of use. Most discussions between contacts in CCGs and the evidence briefing team were informal and rarely involved minuted meetings or formal gatherings of CCG staff. Indeed, we were often responding to requests from one, two or three named individuals who would be leading a piece of work or clinical area on behalf of the CCG as a whole. As such, analysis of records supporting the more formal executive and governing body meetings provided little information about sources used or the decision-making process itself. The ‘unseen and informal spaces’ [35] of decision-making processes, the small numbers of staff involved and the reality that no audit trail existed for sources used during these processes meant that there was little or no ‘traceability’ [36] of use of evidence briefings at an organisational level. Our experience aligns well with others who have faced similar challenges identifying whether systematic reviews are used and the extent to which they add value to decision-making processes in public health [36].

In this study, we sought to add insight as to how much added value the service would offer over alternative or more basic approaches. The evidence briefing service as constituted represented a resource intensive intervention. There was sustained engagement by individuals in the CCGs receiving the evidence briefing service, and because we employed a degree of flexibility in the service delivered (employing a combination of full evidence briefings and shorter more exploratory evidence notes in response to questions raised), we were able to deliver a number of outputs beyond our original. However, impact on explicit instrumental decision-making processes was limited. Whilst we recognise that conceptual use of research to raise awareness and increase knowledge is an entirely appropriate goal in itself, we would question whether this represents a sufficient level of impact to justify sustaining a resource intensive intervention of this type.

The impact of evidence briefings on explicit instrumental decision-making processes was limited to establishing region wide policies relating to interventions of no or low clinical benefit; a process that needed to be both transparent and defendable for participating CCGs. This study may suggest that it is at this meso level where services packaging evidence derived from systematic reviews may most efficiently be deployed to impact on decision-making in a commissioning context. Disinvestment decisions relating to interventions of no or low clinical value remain high on the commissioning agenda. And in our study, established processes to harness research evidence for this type of policy formulation were lacking.

If meso level activity may represent the best focus for resource intensive services, we still need to consider how to systematise research use among individual CCGs. The SPIRIT Action Framework hypothesises that a catalyst is required for the use of research, the response to which is determined by the capacity of the organisation to engage with available research [37]. Where there is sufficient capacity, a series of research engagement actions might occur that facilitate research use. The Framework predicts that the greater the organisational capacity, the more research engagement actions (accessing and appraising research, generating new research and interacting with researchers) will occur which will in turn result in a greater use of research evidence. Using the Framework to reflect on this study, we had catalysts and engagement opportunities (around the questions raised and the briefings produced), but the service as constituted did little to enhance the capacity of the organisation to engage with research. Both baseline and follow-up data suggest that commissioners are well intentioned ad hoc users of research evidence and that they work in a setting where there is a lack of systems and processes to do this routinely. This suggests a knowledge and skills gap that this study has not addressed. The evidence briefing team offered training on how to acquire, assess, adapt and apply evidence to CCGs receiving the evidence briefing service or intervention B. Training could have in part addressed these knowledge and skills gaps, but the offers were not taken up. Rather than making training a demand-led ‘offer’, it may have been better to identify the capacity for research use of each CCG at the outset, and then to provide training relevant to their current state. At the very least, this study has highlighted the importance of building organisational capacity as a component of evidence use, an area that appears to be under researched [38].

Public health specialists have traditionally supported and facilitated the use of research evidence in a commissioning context [39–41]. Throughout this study, we observed that despite its relocation to local government, public health specialist remained accessed by CCGs despite being no longer central to decision-making processes. Some senior staff in participating CCGs had much prior experience of support from public health teams under previous commissioning arrangements. And as the interventions followed soon after the preceding arrangements had ceased, it is perhaps no surprise that the CCG commissioning staff made use of the service offered by CRD. Nevertheless, all the CCGs continued to place value on the knowledge and expertise of trusted ‘critical friends’ [42] in the shape of public health consultants. They provided a bridge between the old and new commissioning arrangements and brought valuable insights and networks from beyond the boundaries of the CCG. Although we often observed commissioners looking out and undertaking fact finding trips to see what other CCGs around the country were doing, the same individuals were often unaware that colleagues in adjacent areas were undertaking similar work or grappling with similar questions. Public health specialists were the individuals viewed most likely to fill this local knowledge gap and to mitigate against a general dissatisfaction with the knowledge-sharing capabilities of the formal Commissioning Support arrangements. Whether fair or otherwise, there was a general perception among CCG informants that the Commissioning Support lacked the necessary infrastructure and or expertise to efficiently acquire, assess and adapt research for use in decision-making. The one-to-one transactional arrangements Commissioning Support had with CCGs were themselves viewed as a barrier to wider knowledge sharing across the region. This danger of ‘network closure’ undermining local knowledge sharing and historically trusted relationships has been anticipated previously [43].

Wye and colleagues have argued that researchers need to build relationships and engage with commissioners locally using commissioners’ preferred methods of conversations and stories, to find out what is wanted and how best to deliver it [41]. Whilst we do not discourage the cultivation of these relationships, the reality of the decision-making process is that any engagement is resource intensive and so researchers need to carefully consider how best to target those relations that will deliver the best return. Somebody needs to be around or ‘in the room’ when ideas first germinate, to spot the potential catalysts to research use and to question what is the evidence for this? Why do we want to pursue this course of action? Given this, Wye et al.’s suggestion that researchers cultivate relationships with local public health teams could represent the intermediary channel through which the use of research by individual CCGs can be influenced [41]. Public health staff are more likely to be ‘in the room’ and have the necessary skills and local networks to facilitate knowledge sharing within and across commissioning landscape. The current emphasis on innovation and the development of new models of health and social care is favouring coproduction approaches to the design, commissioning and delivery of services. This shift may strengthen the intermediary role of public health. But if this intermediary role is to be sustained, public health specialists will need to be supported and resourced to return to playing a more central role in commissioning.

Alongside capacity building and engagement, macro level intervention is also needed to enhance research use at the level of the individual CCG. The Health and Social Care Act mandates research use as a core consideration but, whereas the current policy climate explicitly incentivises innovation and integration, there is no equivalent incentive for finding and applying research to support the many decisions required to turn this vision into a reality. The CCG Assurance Framework focuses on leadership, financial and performance management, planning and delegated functions but contains no specific metrics on whether CCGs are fulfilling their statutory duties in respect of use of evidence obtained from research [44]. If we are serious about shifting CCGs from being well intentioned but inconsistent users of research evidence then a more explicit set of requirements may be necessary. Ideally, the incentive structure that exists for health service innovation and integration may need to be replicated to support CCGs fulfilment of their statutory duties in respect of the use of research under the Act. Without this, the current ad hoc engagement with research is likely to remain.

We are conscious that our findings relate to a specific decision-making context and setting and have been generated at time when the commissioning arrangements are rapidly evolving. Given this, further comparative evaluation and clarification of the role and value of similar demand-led evidence briefing services in other context and settings may be warranted. The SPIRIT Action Framework may provide a guide upon which the evaluation of any future services seeking to increase the use of research in policy can be based [37].

## Conclusions

Access to a demand-led evidence briefing service as constituted in this study did not improve the uptake and use of research evidence by NHS commissioners compared with less intensive and less targeted alternatives. Our study suggests commissioners are well intentioned but lack access to the necessary skills and infrastructure to make use of research evidence routine.
